# Resection of supratentorial brain metastases with intraoperative radiotherapy. Is it safe? Analysis and experiences of a single center cohort

**DOI:** 10.3389/fsurg.2022.1071804

**Published:** 2022-12-26

**Authors:** Philipp Krauss, Kathrin Steininger, Stefan Motov, Bjoern Sommer, Maximilian Niklas Bonk, Abraham Cortes, Christina Wolfert, Georg Stueben, Ehab Shiban, Klaus Henning Kahl

**Affiliations:** ^1^ Department of Neurosurgery, University Hospital Augsburg, Augsburg, Germany; ^2^ Department of Radiooncology, University Hospital Augsburg, Augsburg, Germany

**Keywords:** brain metastasis (BM), neurosurgery, neurooncology, intraoperative radiotherapy (IORT), complications

## Abstract

**Introduction:**

Intraoperative Radiotherapy (ioRT) is an emerging treatment option in oncologic surgery for various diseases including intraaxial brain lesions to improve surgical outcome and accelerate the adjuvant oncologic therapy. Despite its use in glioma surgery, the application and data regarding ioRT in the treatment of brain metastases (BMs) is sparse. Here were report the largest series of supratentorial BMs treated with resection and ioRT according to functional outcome and adverse events.

**Methods:**

We performed a retrospective chart review analysis of patients undergoing surgery for BMs following an interdisciplinary tumor board decision in every case with ioRT at our institution. Patient properties, functional status (Karnofsky Performance Score/KPS) before and after surgery as well as oncologic (disease, recursive partitioning analysis, lesion size) and operative parameters were analyzed until hospital discharge. Adverse events (AE) were recorded until 30 days after surgery and rated according to the Clavien Dindo Grading (CDG) scale.

**Results:**

70 patients (40 female) with various oncologic diseases were identified and analyzed. Six underwent prior RT. Mean age was 66 ± 11 years. Preoperative median KPS was 80% with a mean BM volume of 3.2 ± 1.2 cm^3^. Nine patients (13%) experienced in total 14 AEs, including 2 cases (3%) of postoperative death (CDG5) and 2 with new postoperative epilepsy necessitating additional pharmacotreatment (CDG2). Five patients suffered from new neurologic deficit (CDG1) not needing further surgical or medical treatment. After surgery, the neurological status in 7 patients (10%) deteriorated while it improved in 21 cases (30%). Patients experiencing AEs had longer hospitalization and poorer postoperative KPS mdn. 90 vs. 80%. There was no statistically significant deterioration of the functional status during the immediate postoperative course in the whole patient cohort.

**Conclusion:**

Surgery for supratentorial BMs with ioRT seems safe and feasible. Further studies on the benefit regarding oncologic outcome need to be performed.

## Introduction

Intraoperative radiotherapy (ioRT) has proven its applicability in various diseases especially breast and colorectal cancer surgery. The goal is a quick transition from operation to adjuvant (radiation and/or systemic) therapy, which otherwise can be delayed by wound healing issues after surgery. In Neurooncology, adjuvant radiotherapy is standard of care for most malignant neoplasms. Most experiences exist for high grade gliomas (HGG), in which ioRT is used to focally reach higher isodoses as a boosting approach. Though, for the treatment of brain metastases (BMs), little evidence on the applicability, treatment effects and risks exists to date. However, especially BM patients might benefit from a quick transition to adjuvant therapy to tackle the systemic disease. This includes the avoidance absence of surgical site infections (SSIs) and the possible positive impact of reduced application of corticosteroids compromising new molecular oncologic therapies. Therefore, ioRT for BMs might possibly offer benefits in the comprehensive treatment strategy.

The aim of this study is to evaluate the applicability of ioRT for supratentorial BMs emphasizing surgical aspects, the rate of adverse events (AEs) and short term functional outcome. To our knowledge, this single center cohort represents the largest series to be reported to date.

## Methods

### Ethics approval

The study protocol was approved by the local ethics committee (UKA/LMU) in accordance to the Declaration of Helsinki. For this retrospective observational study, no individual informed consent was necessary according to the ethics committee's guidelines and regulations.

### Study design

We performed a retrospective analysis of patient-specific clinical records in one single tertiary neurosurgical center. The analyzed parameters included age, sex, Karnofsky Performance Scale (KPS) before and after surgery as well as KPS difference (pre/post surgery), the recursive partitioning analysis (RPA) for BMs, length of surgery (LOS), length of radiation (LORT), volume of the metastasis, volume of radiation applicator, radiation dose, total number of brain metastases, length of hospitalization (LOH) and adverse events during hospitalization according to the Clavien-Dindo Grading system (CDG) (1–3). Furthermore, the histopathology of the underlying neoplasm was analyzed.

### Patient selection

Electronic data files of all adult patients who underwent resection of supratentorial intraaxial brain metastases and received ioRT between 2014 and 2022 were screened. Patients <18 years of age and patients that underwent resection in the posterior fossa were excluded from the analysis.

### Intraoperative radiotherapy

Indication for treatment was confirmed by the local multidisciplinary tumor board in all cases. Between 2014 and 2017 ioRT was offered to the patient based on interdisciplinary consensus on an individual basis. From 2017 on, it was offered routinely as an alternative to postoperative external-beam RT following an expert panel of the German Society for Radiation Oncology (DEGRO) guideline [Expert panel decision DEGRO, inquiry 123, 17.02.2017]. Patients were considered ineligible if (1) the distance between the border of the MRI contrast-enhancing lesion and the brainstem was <5 mm, (2) there was a history of small-cell lung cancer or (3) the resection trajectory was estimated to not allow a safe introduction of the radiation applicator. All patients signed informed consent for resection and ioRT. After tumor exstirpation, the resection cavity was irradiated with 50-kV x-rays *via* an INTRABEAM system (ZEISS MEDITEC AG, Oberkochen, Germany). The device and procedure have been described previously (4). A suitable spherical applicator was installed according to the size of the resection cavity, providing direct contact of the cavity walls to the surface of the applicator. Radiation dose was prescribed to the surface of the applicator corresponding to the target volume/dose concept of postoperative SRS cavity treatment (GTV = CTV = cavity). During the perioperative course, steroids were administered orally following a local standard operating procedure.

### Statistics

Statistical analysis was performed using the software SPSS Statistics™ (version 25, IBM Corp, Armonk, New York, United States). Normal distribution was assumed for continuous data according to the central limit theorem. An unpaired 2-tailed student's *t*-test was used to compare the significance of means between two groups. Pearson's or Spearman's correlation was used respectively. Ordinal data was analyzed with an unpaired Mann–Whitney *U*-test, dichotomous by means of *χ*^2^-test. Data in text and graphs are shown as mean and standard deviation (SD) for continuous data and as median and interquartile range for ordinal data. A *p* value ≤ .05 was considered significant and indicated by “*”, *p* values ≤ .01 were indicated by “**,” and values ≤ .001 by “***.”

## Results

### Patient population

In this study, 70 patients (40 female) were identified and met the inclusion criteria. Mean age was 65.6 ± 11.2 years with an adequate functional status [mdn. KPS 80% (80%–90%) IQR]. Six patients had prior cranial percutaneous stereotaxic RT for other focal brain lesions (for further baseline characteristics see Table 1).

**Table 1:** Baseline characteristics: y, years; f/m, female/Male; KPS, Karnofsky performance score; Pre, preoperative; RPA, recursive partitioning analysis; y/n, yes/no; BM, brain metastasis; F, frontal; T, temporal; *P*, parietal; O, occipital; *n*, number; RT, radiotherapy; Gy, gray; LOS, length of surgery; LORT, length of radiotherapy; post, postoperative; AE, adverse event; LOH, length of hospitalization; d, days; NSCLC, non-small cell lung cancer; SCC, squamous cell cancer; RCC, renal cell cancer; SNC, sinunasal cancer; data is shown as [mean ± SD/median (interquartile range)].

	Total (*n* = 70)
**Baseline Characteristics**	
Age (y)	65.6 ± 11.2
Sex (f/m)	40/30
KPS Pre (%)	80 [80–90]
RPA	2 [2–2]
Neurological deficit (y/n)	42/18
BM localisation (F/T/*P*/O)	27/12/11/20
BM localisation (central/left/right)	0/35/35
BM size (cm^3^)	3.2 ± 1.2
Total BM (*n*)	1.7 ± 1.1
Prior RT (y/n)	6/64
**Surgery**	
Applicator size (cm^3^)	2 [2–2.5]
RT Dose (Gy)	20 [20–20]
LOS (min)	153 ± 44
LORT (min)	10 ± 4
**Postoperative Outcome**	
KPS Post (%)	80 [80–90]
KPS *Δ* (%)	0 [0–0]
Neurological deficit deteriorated (y/n)	7/63
Neurological deficit improved (y/n)	21/49
Patients with AE (y/n)	9/61
AE total (*n*)	14
LOH (d)	7.8 ± 4.3
**Oncologic Disease**	
NSCLC (Adeno)	21
NSCLC (SCC)	2
NSCLC (other)	5
Gastrointestinal (Adeno)	8
Gastrointestinal (SCC)	1
Malignant Melanoma	12
Breast Cancer	7
RCC	4
SNC	2
Parotid (Adeno)	2
Ovarian Cancer	2
Urothelial Carcinoma	1

### Surgery and outcome

Mean LOS was 153 min including a mean LORT of 10 min. The median radiation dose applied was 20 Gy (13.4 Gy: *n* = 1; 16 Gy: *n* = 2, 18 Gy: *n* = 6, 20 Gy: *n* = 63). The median diameter of the radiation applicator was 2 cm [2 cm–2.5 cm] IQR. After surgery, the mean LOH was 7.8 days. In the whole cohort, patients had a median postoperative KPS of 80% with no substantial decline in the functional status (median decline 0% KPS). Improvement of a preoperative neurological deficit occurred in *n* = 21 patients. In these patients median KPS improved from 80% before to 90% after surgery. A new neurological deficit or worsening of a preexistent deficit occurred in *n* = 7 patients resulting in a reduction of median KPS from 90% before surgery to 70% after surgery. After surgery two patients had a decline in functional status possibly delaying systemic therapy (KPS 70% to 60%, KPS 90% to KPS 0%) and two patients had an improvement in functional status respectively (both KPS 40% to 70%). All other patients remained unchanged regarding their functional status (for further characteristics see Table 1). High RPA values significantly inversely correlated with the functional status after (KPS post; *r* = −.52; *p* < .01) surgery, but not with changes in functional status (KPS *Δ*; *r* = .16; *p* > .05). Further, high preoperative functional status correlated significantly with postoperative functional outcome (KPS post; *r* = .81; *p* < .01) and inversely with change in KPS (KPS *Δ*; *r* = −.35; *p* < .01). No further significant correlations were found (Table 2).

**Table 2:** Functional outcome correlation (spearman correlation): y, years; KPS, Karnofsky performance score; Pre, preoperative; post, postoperative; RPA, recursive partitioning analysis; BM, brain metastasis; *n*, number; RT, radiotherapy; Gy, gray; LOS, length of surgery; LORT, length of radiotherapy; n.s., non-significant; data is shown as spearman's correlation coefficient R with level of significance *p*.

Corellation	KPS Post	KPS *Δ*
Age (y)	*R* = −.18; n.s.	*R* = .12; n.s.
KPS Pre (%)	***R* = .81; *p* < .01**	***R* = −.35; *p* < .01**
KPS Post (%)	**–**	*R* = .15; n.s.
KPS *Δ* (%)	*R* = .15; n.s.	–
RPA	***R* = −.52; *p* < .01**	*R* = .16; n.s.
Total BM (*n*)	*R* = −.21; n.s	*R* = .16; n.s.
BM size (cm^3^)	*R* = −.13; n.s.	*R* = .19; n.s.
Applicator size (cm^3^)	*R* = .02; n.s.	*R* = .09; n.s.
RT Dose (Gy)	*R* = .02; n.s.	*R* = −.02; n.s.
LOS (min)	*R* = −.12; n.s.	*R* = .04; n.s.
LORT (min)	*R* = .01; n.s.	*R* = .01; n.s.

### Adverse events

In this study, 9/70 patients experienced in total 14 AEs within 30 days after surgery resulting in 7 deteriorations of the neurological status (2 resulting in deterioration of KPS ≤60% from better prior levels) (Table 3). Patients suffering from AEs had significantly lower postoperative functional status and longer LOH (Table 4). No further significant differences were found to be attributed with AEs in this cohort (Table 4).

**Table 3:** Adverse events: data is shown for each individual patient (who experienced an AE) as adverse event including corresponding grading according to the clavien dindo grading system.

	Complications	Clavien Dindo Grading
Patient 6	Hemiparesis	1
Patient 14	Dysphasia	1
Patient 27	Seizure	2
Patient 34	Hemiparesis	1
Patient 36	Renal dysfunction, urinary tract infection, pneumonia, death	1, 2, 4a, 5
Patient 44	Seizure	2
Patient 51	Hemiparesis	1
Patient 66	Surgical site infection, pneumonia, ventriculitis, cerebral ischemia, death	2, 2, 2, 3b, 5
Patient 69	Hemiparesis	1

**Table 4:** Comparison patients with/without AE: y, years; f/m, female/Male; KPS, Karnofsky performance score; Pre, preoperative; post, postoperative; RPA, recursive partitioning analysis; y/n, yes/no; BM, brain metastasis; *n*, number; RT, radiotherapy; Gy, gray; LOS, length of surgery; LORT, length of radiotherapy; AE, adverse event; LOH, length of hospitalization; d, days; data is shown as [mean ± SD/median (interquartile range)] with level of significance *p*.

	Patients with AE (*n* = 9)	Patients without AE (*n* = 61)	*p*-value
Age (y)	64.4 ± 8.9	65.3 ± 11.5	.82
sex (f/m)	4/5	36 /25	.41
KPS Pre (%)	80 [80–90]	80 [80–90]	.99
KPS Post (%)	80 [30–80]	90 [80–90]	**<.01**
RPA	2 [2–2]	2 [2–2]	1
Total BM (*n*)	1.4 ± 0.7	1.7 ± 1.1	.51
BM size (cm^3^)	2.5 ± 0.9	3.3 ± 1.3	.08
Applicator size (cm^3^)	2.1 ± 0.5	2.4 ± 0.7	.29
RT Dose (Gy)	2 [1.5–2.5]	2.5 [2–3]	.91
LOS (min)	157 ± 72	154 ± 39	.84
LORT (min)	10 ± 4	10 ± 4	.61
LOH (d)	12 ± 8	7 ± 3	**<.001**

## Discussion

In this study we evaluated the operative parameters and short-term outcome of patients undergoing microsurgical resection of supratentorial BMs with ioRT. To our knowledge, this is the largest cohort to systematically report ioRT in supratentorial BMs.

### Baseline parameters

In this cohort, the patients had an adequate functional status mdn. KPS 80% but only 18/70 patients were without neurologic alteration. In 42/70 patients, single BMs were operated and only 6/70 had prior cranial RT. This represents a “classic” population for BM surgery with regularly symptomatic BM, good functional status but low BM burden and is comparable to prior cohorts on ioRT for BMs (5–8). The main oncologic disease was NSCLC, followed by malignant melanoma, gastrointestinal and breast cancer. In prior reports similar distribution of oncologic entities was reported. Whether these BM respond differently to ioRT remains unclear to date due to yet small samples and needs to be analyzed in future studies.

### Surgical/ioRT parameters

In this cohort a mdn. radiation dose of 20 Gy, was applied for a mean time of 10 ± 4 min. In prior reports, various resective strategies have been reported ranging from biopsy to microsurgical resection (5–7). Further, different radiation doses applied using different radiation devices ranging from 14 Gy to 30 Gy (5–7). To which extent these variances influence the surgical work flow and postoperative AEs cannot be reconstructed as most reports do not focus on the surgical perspective.

### Functional outcome

The utmost goal of surgery for BMs is to preserve or even improve the functional status as it is known to influence the longer-term outcome and survival (9). In this cohort, most patients started from a good functional KPS with 21/70 patients showing improvement or relief of prior neurological deficits, while 7 patients experienced a worsening or new neurological deficit. The median functional status did not change in our cohort and remained at KPS 80%. We found postoperative KPS to be positively related to preoperative KPS possibly attributing only minor burden due to the surgical intervention. The mdn. change in KPS inversely correlated with the preoperative functional status indicating which statistically might be influenced by a ceiling effect as KPS of 100% does by definition leaves no room for improvement. Furthermore, our analysis shows, that surgery with ioRT is equally safe regarding the patients age or BM/resection cavity size. The only other study of microsurgical resection with ioRT reporting direct perioperative functional outcome showed a mdn. slight improvement from KPS 80% to KPS 90% (6). In this study, slightly different RT settings were chosen (14 Gy). Only one patient experienced functional deterioration at the one month follow up. The mdn. overall survival was 36 months with a 2.5% radionecrosis rate compared to 26 months reported in another contemporary series with zero radionecrosis (4). Therefore, contemporary data, including this study supports the hypothesis, that ioRT in microsurgical resection for BMs does not influence the functional outcome in a negative way, enabling a quick progress to systemic therapy.

### Adverse events

Overall adverse event rate was 13% with 9 patients experiencing 14 AEs. AE rates in BM surgery were reported to be between 13%–19% with mortality rates of 4% and SSI rates of 0.7%–2%. New neurological deficits are reported to occur in 8%–18% which is mainly dependent on the eloquence of the BM location (10–12). In a prior report on ioRT for supratentorial metastasis a complication rate of 11% was reported (13). Absence of AEs is of utmost importance to avoid delay in systemic therapy. Most of them were low grade AEs according to the CDG (1 and 2) not necessitating surgical intervention. One patient experienced a SSI with concomitant ventriculitis and death reflecting a SSI Rate of <2%. As percutaneous RT might influence wound conditions, this is not indicated for ioRT in our cohort (14, 15).

### Future perspectives for intraoperative radiotherapy in surgery for brain metastasis

Intraoperative radiotherapy is increasingly applied in oncologic surgery including HGG and BMs (4, 7, 16). Whether it improves local control and prevents leptomeningeal disease remains unclear. Further interest has grown on neoadjuvant RT for BMs to reduce the risk of leptomeningeal disease (17, 18). Furthermore, radiation of BMs might create an immune reaction by altering the blood brain barrier and exposing neoplastic tissue (19). Whether neoadjuvant RT alters the molecular profile of BMs is unknown and tissue for molecular analysis before RT cannot be analyzed. If ioRT might enhance immune reactions, while keeping the possibility to harvest “fresh” native tissue will need further investigations. Furthermore, regarding the increasing use of immunotherapy in oncology, the regular use of steroids during the postoperative course, when percutaneous RT is performed, might be quickly reduced in the case of ioRT. This might enable a faster transfer to systemic therapy including immunotherapy without the compromising effect of steroids. Apart from the influence on local control, the appearance of radionecrosis is a challenging condition in brain tumor surgery and radiation. Even though, radionecrosis has to be regarded distinct from neoplastic relapse, its aggressive nature can cause tissue damage, neurological symptom burden and might be regarded as neoplastic like lesion. The data published to date indicate a reduced rate of radionecrosis in patients undergoing ioRT for BMs (4).

Regarding the AE profile of RT, the paradigm has shifted from whole brain radiotherapy to more focal radiotherapy in the treatment of single BMs due to its more favorable outcome regarding neurocognitive function and quality of life (20). The potential impact on neurocognitive function using ioRT in the treatment of supratentorial metastases need to be systematically investigated in a larger cohort with long term follow up.

If ioRT represents a more cost-effective alternative to classic adjuvant radiotherapy, cannot be answered from this study. In breast cancer surgery, where ioRT is used more extensively, cost-effectiveness remains controversial (21, 22). Furthermore, comparing breast cancer surgery from neurooncological interventions is hard, as surgical as well as radiation parameters (affecting the possible prolongation or the LOS) have important differences. While the costs of reusable ioRT systems can be regarded as business case, the infrastructure to perform ioRT largely depends on the cooperation of an experienced interdisciplinary team, which might not be present in every center. Further cost-effectiveness analysis, respecting the variety of international healthcare systems, are certainly needed.

### Study limitations

This study has several limitations, that have to be clearly addressed. First, the retrospective nature of the study is inherently prone to selection bias. In our neurooncological unit every case is discussed in a multidisciplinary oncologic board. At our center ioRT is performed for several years on a regular basis and after confirmation of the indication, the patient informed about the possibility of ioRT.

The oncologic disease treated included various entities. As the main interest of this study was to evaluate the feasibility and perisurgical outcome, we claim that this variety is negligible, especially as the functional performance prior to surgery was homogenous. Whether our findings ultimately affect the oncologic prognosis cannot be answered based on this data. Nevertheless, as the prognostic factor of functional outcome is an established parameter, our findings implicate a favorable outcome if surgery combined with ioRT is performed.

## Conclusion

In this retrospective study, we report the largest cohort of patients undergoing resection for supratentorial brain metastases with intraoperative radiotherapy. The operative parameters, functional outcome and adverse events are reported and do not indicate inferiority to previously published reports of resection followed by percutaneous radiotherapy. The safety of this technique is demonstrated and ioRT should be considered an option in the surgery of brain metastases.

## Data Availability

The raw data supporting the conclusions of this article will be made available by the authors, without undue reservation.
